# A randomized clinical trial using cyclopentolate and tropicamide to compare cycloplegic refraction in Chinese young adults with dark irises

**DOI:** 10.1186/s12886-021-02001-6

**Published:** 2021-06-10

**Authors:** Ruxia Pei, Zhuzhu Liu, Hua Rong, Liqiong Zhao, Bei Du, Na Jin, Hongmei Zhang, Biying Wang, Yi Pang, Ruihua Wei

**Affiliations:** 1 Tianjin Key Laboratory of Retinal Functions and Diseases, Tianjin Branch of National Clinical Research Center for Ocular Disease, Eye Institute and School of Optometry, Tianjin Medical University Eye Hospital, 251 Fukang Road, Nankai District, Tianjin, China; 2 Illinois College of Optometry, 3241 S, Michigan Ave, Chicago, IL 60616 USA

**Keywords:** Chinese young adult, Tropicamide, Cyclopentolate, Autorefraction, Subjective refraction

## Abstract

**Background:**

To evaluate the necessity of cycloplegia for epidemiological studies of refraction in Chinese young adults (aged 17–22 years) with dark irises, and to compare the cycloplegic effects of 1% cyclopentolate and 0.5% tropicamide in them***.***

**Methods:**

A total of 300 young adults (108 males and 192 females) aged 17 to 22 years (mean 19.03 ± 1.01) were recruited from Tianjin Medical University from November 2019 to January 2020. Participants were randomly divided into two groups. In the cyclopentolate group, two drops of 1% cyclopentolate eye drop were administrated (one drop every 5 min), followed by autorefraction and subjective refraction 30 to 45 min later. In the tropicamide group, four drops of 1% Mydrin P (Tropicamide 0.5%, phenylephrine HCl 0.5%) eye drop were given (one drop every 5 min), followed by autorefraction and subjective refraction 20 to 30 min later. The participants and the examiners were masked to the medication. Distance visual acuity, intraocular pressure (IOP), non-cycloplegic and cycloplegic autorefraction (Topcon KR-800, Topcon Co. Tokyo, Japan), non-cycloplegic and cycloplegic subjective refraction and ocular biometry (Lenstar LS-900) were performed.

**Results:**

The values of spherical equivalent (SE) and sphere component were significantly different before and after cycloplegia in the cyclopentolate group and the tropicamide group (*p* < 0.05). The mean difference between noncycloplegic and cycloplegic autorefraction SE was 0.39 D (±0.66 D) in the cyclopentolate group and 0.39 D (±0.34 D) in the tropicamide group. There was no significant difference in the change of SE and sphere component after cycloplegia between the cyclopentolate group and the tropicamide group (*p* > 0.05). In each group, no significant difference was found between autorefraction and subjective refraction after cycloplegia (*p* > 0.05). We also found that more positive or less negative cycloplegic refraction was associated with the higher difference in SE in each group.

**Conclusions:**

Cycloplegic refractions were generally more positive or less negative than non-cycloplegic refractions. It is necessary to perform cycloplegia for Chinese young adults with dark irises to obtain accurate refractive errors. We suggest that cycloplegic autorefraction using tropicamide may be considered as a reliable method for epidemiological studies of refraction in Chinese young adults with dark irises.

**Trial registration:**

The study was registered on September 7, 2019 (Registration number: ChiCTR1900025774).

## Background

Cycloplegic refraction using cycloplegic agents is an effective way to control accommodation [1–3]. Adequate cycloplegia is of great importance to obtain accurate refractive errors [2, 4–7]. Cycloplegic refraction is the gold standard method for epidemiological studies in children and adolescents [4, 8], but the use of cycloplegia in young adults is still controversial. Some studies have been conducted to determine the necessity of cycloplegia and different results have been reported in young adults [9–13]. Yun-Yun Sun et al. suggested that cycloplegia is essential and necessary for Chinese young adults (mean aged 20.2 ± 1.5 years) in epidemiological studies [9]. The Tehran Eye study [12] found that cycloplegia is required for epidemiological studies, up to the age of 50. On the contrary, Sanfilippo et al [13] and Krantz et al [11] suggested that it is not necessary to perform cycloplegia in young adults for epidemiological studies of refraction.

The three most commonly used cycloplegic agents include atropine, tropicamide and cyclopentolate [14]. The ideal cycloplegic agent characterized by rapid onset, short duration of action, complete cycloplegia, and absence of side effect [15]. Atropine is the gold standard for its cycloplegic effect, but the onset is very slow and recovery time is always as long as 15 to 20 days [16]. Therefore, it is not routinely used as a diagnostic agent in adults. Cyclopentolate is a synthetic antimuscarinic cycloplegic agent which is widely accepted as the cycloplegic agent in children and has been showed as effective as atropine at obtaining cycloplegia [17]. For individuals with dark irises, cyclopentolate is characterized by an onset of quick action (30–45 min), a relatively short duration of action (24–48 h) and few side effects [18]. Tropicamide is a synthetic analog of tropic acid which is another choice of cycloplegic agent, also known as a rapid and safe agent for cycloplegic refraction [19]. Compared with cyclopentolate, tropicamide is more acceptable in patients [20, 21] for its profiles of faster onset (20–30 min) and recovery (6–7 h).

For young adults, little research has been done to compare the cycloplegic effects of cyclopentolate and tropicamide [20–22]. Gettes et al [22] and Hofmeister et al [21] suggested that cyclopentolate and tropicamide may be equally effective in refractive measurements in young adults. One study of 25 Black young adults with dark irises found that the cycloplegic effect of cyclopentolate was stronger than tropicamide [20]. It has been reported that there is a delay in onset and a decrease in magnitude of cycloplegic effect with increased iris pigmentation in eyes caused by pigment binding of the cycloplegic agents [23, 24]. Manny et al [18] found it took longer for adults with dark irises to reach the maximum cycloplegia effect. However, there is a lack of comparable study on Chinese young adults (aged 17–22 years) with dark irises.

The purpose of this prospective randomized controlled study was to evaluate the necessity of cycloplegia in Chinese young adults (aged 17–22 years) with dark irises, and to compare the cycloplegic effects of cyclopentolate and tropicamide in them.

## Method

### Subjects

Young Chinese adults aged 17 to 22 years with dark irises were recruited from Tianjin Medical University from November 2019 to January 2020. Written informed consent was obtained from each participant before the data collection. This study was approved by Tianjin Medical University Eye Hospital ethics committee and the conduct of the study adhered to the tenets of the Declaration of Helsinki.

Participants with strabismus, amblyopia, nystagmus, glaucoma, contact lens, a history of ocular surgery, trauma or other ophthalmic diseases were excluded from the study.

### Procedures

Considering 80% power and a 5% confidence level and allowing for 10% loss to follow-up, a sample size of 150 young adults in each of the two intervention arms was sufficient. The randomization number was generated automatically by the manager of the Clinical Trial Coordination Centre by means of a computerized random-number generator. The intervention group was concealed in numbered, opaque envelopes which were contained in a box. Each young adult selected one envelope and gave it to the nurse, who opened the envelope and administered the appropriate cycloplegic agent at that time. Participants were randomly divided into two groups according to the numbers. The cyclopentolate group performed a cyclopentolate regimen with the administration of one drop of 0.4% oxybuprocaine hydrochloride followed by two drops of 1% cyclopentolate hydrochloride (Alcon) at 5-min intervals. Cycloplegic autorefraction and subjective refraction were performed between 30 min and 45 min after the last instillation of cyclopentolate, when the pupillary light reflex was eliminated. The tropicamide group performed a tropicamide regimen with the administration of one drop of 0.4% oxybuprocaine hydrochloride followed by four drops of Mydrin P (Tropicamide 0.5%, phenylephrine HCl 0.5%; Santen Pharmaceutical, Shiga, Japan) at 5-min intervals. Cycloplegic autorefraction and subjective refraction were performed between 20 min and 30 min after the last instillation of Mydrin P, when the pupillary light reflex was eliminated. To prevent discomfort in participants, a drop of oxybuprocaine eye drops, had been used before administration of cycloplegic agent. The cycloplegic agent was carefully instilled into the conjunctival sac in order to avoid irritating tearing, which may affect the cycloplegic function [25]. The examiners and participants were masked which cycloplegic agent was applied.

### Examinations

All participants underwent a comprehensive standard examination at the optometry laboratory in Tianjin Medical University. Distance visual acuity (logMAR chart), intraocular pressure (non-contact tonometry, Topcon CT-1), non-cycloplegic and cycloplegic autorefraction (Topcon KR-800), non-cycloplegic and cycloplegic subjective refraction and ocular biometry (Lenstar LS-900) were performed. All ophthalmologic examinations were conducted by one ophthalmologist, six optometrists and two nurses. A training course was conducted to ensure all examinations would be performed under the same criteria and a comprehensive standard procedure was made for the whole outcome recording during the study. Uncorrected visual acuity (UCVA) and best corrected visual acuity (BCVA) was measured using logMAR chart with tumbling E. Intraocular pressure (IOP) before cycloplegia was measured using non-contact tonometry (Topcon CT-1). Autorefraction and subjective refraction were measured by the same optometrists before and after cycloplegia. Subjective refraction was performed based on subjective refinement of the autorefractor readings until best-corrected visual acuity was achieved (BCVA).

### Definitions

The noncycloplegic and cycloplegic refraction obtained from the autorefractor and subjective refraction between the two groups were decomposed into three components: sphere (S), cylinder (C) and axis (A). SE was calculated according to using the formula: SE = S + C/2. In this study, young adults were divided into five categories based on cycloplegic SE: low myopia (− 3.0D < SE ≤ − 0.5D), moderate myopia (− 6.0D < SE ≤ − 3.0D), high myopia (SE ≤ − 6.0D), emmetropia (− 0.5D < SE ≤ + 0.5D) and hyperopia (SE > + 0.50D). The hyperopic participants were all categorized in one group because of the low incidence of hyperopia in young adults.

### Statistical analysis

Results from autorefraction and subjective refraction before and after cycloplegia were recorded. As the correlation coefficients of non-cycloplegic refraction and cycloplegic refraction between OD and OS were high, only data from right eyes were used for data analysis. Numeric values were presented as mean ± standard deviation (SD) or median. Independent-samples T test, chi-square test and Wilcoxon test were applied to compare basic participants characteristics and differences in refraction (spherical and cylinder components, and SE) after cycloplegia. Pearson’s correlation coefficient was used to show the correlation between cycloplegic autorefraction and cycloplegic subjective refraction. Spearman correlation analysis was used to determine the relationship between difference in SE and cycloplegic SE because those measurements were not normally distributed. A two-sided *p* value less than 0.05 was considered statistically significant. Statistical analysis was performed using a computer package program SPSS 23.0 (IBM Co., Armonk, NY).

## Results

A total of 300 young adults (108 males and 192 females) aged 17 to 22 years (mean 19.03 ± 1.01y) were ***included*** in this study. Of the 300 young adults, 150 received cyclopentolate and 150 received tropicamide for cycloplegia. None of them was lost to follow up. No significant differences were observed in age, gender, and non-cycloplegic refraction between the cyclopentolate group and the tropicamide group (Table 1).

**Table 1:** Participant’s characteristics

	Cyclopentolate (*n* = 150)	Tropicamide (*n* = 150)	*p*-value
Age (y)	18.95 ± 1.05	19.10 ± 0.98	0.184^*^
Sex			0.463^☆^
Male	57(38.0%)	51(34.0%)	
Female	93(62.0%)	99 (66.0%)	
Mean autorefractive error (D)			
Sphere (noncycloplegic)	−4.01 ± 2.26	−3.98 ± 2.61	0.923^*^
Cylinder (noncycloplegic)	−0.77 ± 0.96(− 0.50)	−0.83 ± 0.77(− 0.50)	0.101^+^
Spherical equivalent (noncycloplegic)	− 4.39 ± 2.37	−4.39 ± 2.70	0.999^*^
Mean subjective refractive error (D)			
Sphere (noncycloplegic)	−4.06 ± 2.22	−3.97 ± 2.59	0.744^*^
Cylinder (noncycloplegic)	−0.62 ± 0.78(− 0.50)	−0.71 ± 0.73(− 0.50)	0.053^+^
Spherical equivalent (noncycloplegic)	−4.37 ± 2.31	−4.33 ± 2.64	0.877^*^

Values are presented as mean ± standard deviation, median or number (%)

*D* diopters

*p-value by t-test; ^☆^*p*-value by chi-square test; +*p*-value by Wilcoxon test

Tables 2 and 3 show the results of refractions before and after cycloplegia in the cyclopentolate group and the tropicamide group, respectively. The overall distribution of difference in refraction indicated that cycloplegic refractions were more positive or less negative than non-cycloplegic refractions. The values of SE and sphere component were significantly different before and after cycloplegia in tropicamide group and cyclopentolate group (*p* < 0.05 for all). Table 4 shows the changes in refraction after cycloplegia in the two groups. No significant differences were found in the changes of SE and sphere component between the cyclopentolate group and the tropicamide group (*p* > 0.05). There was no significant difference in the cylinder component before and after cycloplegia.

**Table 2:** Non-cycloplegic and cycloplegic refraction in the cyclopentolate group

	Non-cycloplegic	Cycloplegic	Difference	*p*-value
Autorefraction				
Sphere (D)	−4.01 ± 2.26	−3.60 ± 2.38	0.40 ± 0.34	< 0.01*
Cylinder (D)	−0.77 ± 0.96(− 0.50)	−0.79 ± 0.90(− 0.50)	−0.02 ± 0.27(0.00)	0.311^+^
Spherical Equivalent (D)	−4.39 ± 2.37	−4.00 ± 2.49	0.39 ± 0.36	< 0.01*
Subjective refraction				
Sphere (D)	−4.06 ± 2.22	−3.67 ± 2.38	0.39 ± 0.32	< 0.01*
Cylinder (D)	−0.62 ± 0.78(− 0.50)	−0.61 ± 0.74(− 0.50)	0.02 ± 0.29(0.00)	0.446^+^
Spherical Equivalent (D)	−4.37 ± 2.31	−3.97 ± 2.50	0.40 ± 0.35	< 0.01*

Values are presented as mean ± standard deviation or median

*p-value by t-test; +*p*-value by Wilcoxon test

**Table 3:** Non-cycloplegic and cycloplegic refraction in the tropicamide group

	Non-cycloplegic	Cycloplegic	Difference	*p*-value
Autorefraction				
Sphere (D)	−3.98 ± 2.61	− 3.58 ± 2.76	0.40 ± 0.32	< 0.01*
Cylinder (D)	−0.83 ± 0.77(− 0.50)	−0.84 ± 0.78(− 0.50)	−0.01 ± 0.27(0.00)	0.677^+^
Spherical equivalent (D)	−4.39 ± 2.70	− 4.00 ± 2.86	0.39 ± 0.34	< 0.01*
Subjective refraction				
Sphere (D)	−3.97 ± 2.59	−3.58 ± 2.78	0.39 ± 0.34	< 0.01*
Cylinder (D)	−0.71 ± 0.73(− 0.50)	−0.71 ± 0.77(− 0.50)	0.00 ± 0.31(0.00)	0.543^+^
Spherical equivalent (D)	−4.33 ± 2.64	−3.93 ± 2.87	0.39 ± 0.38	< 0.01*

Values are presented as mean ± standard deviation or median

*p-value by t-test; +*p*-value by Wilcoxon test

**Table 4:** Changes in autorefraction and subjective refraction before and after cycloplegia

	Cyclopentolate Group (*n* = 150)	Tropicamide Group (*n* = 150)	*p*-value^+^
Autorefraction			
Sphere change (D)	0.40 ± 0.34(0.25)	0.40 ± 0.32(0.25)	0.903
Cylinder change (D)	−0.02 ± 0.27(0.00)	−0.01 ± 0.27(0.00)	0.650
Spherical equivalent change (D)	0.39 ± 0.36(0.38)	0.39 ± 0.34(0.38)	0.962
Subjective refraction			
Sphere change (D)	0.39 ± 0.32(0.25)	0.39 ± 0.34(0.25)	0.812
Cylinder change (D)	0.02 ± 0.29(0.00)	0.00 ± 0.31(0.00)	0.844
Spherical equivalent change (D)	0.40 ± 0.35(0.38)	0.39 ± 0.38(0.38)	0.526

Values are presented as mean ± standard deviation(median)

+*p*-value by Wilcoxon test

Results of sphere and spherical equivalent values obtained by cycloplegic autorefraction and cycloplegic subjective refraction are listed in Table 5. Mean sphere and spherical equivalent refraction values were of no significant difference between cycloplegic autorefraction and cycloplegic subjective refraction in cyclopentolate group and in tropicamide group. In the cyclopentolate group, the correlation between the two methods was 0.989 for sphere and 0.983 for spherical equivalent values. In the tropicamide group, the correlation between the two methods was 0.993 for sphere and 0.992 for spherical equivalent values.

**Table 5:** Mean values by cycloplegic autorefraction and cycloplegic subjective refraction

	Autorefraction	Subjective refraction	*p*-value*	r
Cyclopentolate Group (n = 150)				
Sphere (D)	−3.60 ± 2.38	−3.67 ± 2.38	0.744	0.989
Spherical equivalent (D)	−4.00 ± 2.49	−3.97 ± 2.50	0.475	0.983
Tropicamide Group (n = 150)				
Sphere (D)	−3.58 ± 2.76	−3.58 ± 2.78	0.866	0.993
Spherical equivalent (D)	−4.00 ± 2.86	−3.94 ± 2.87	0.532	0.992

Values are presented as mean ± standard deviation

*p-value by t-test. r by Pearson correlation analysis

**Fig. 1:**
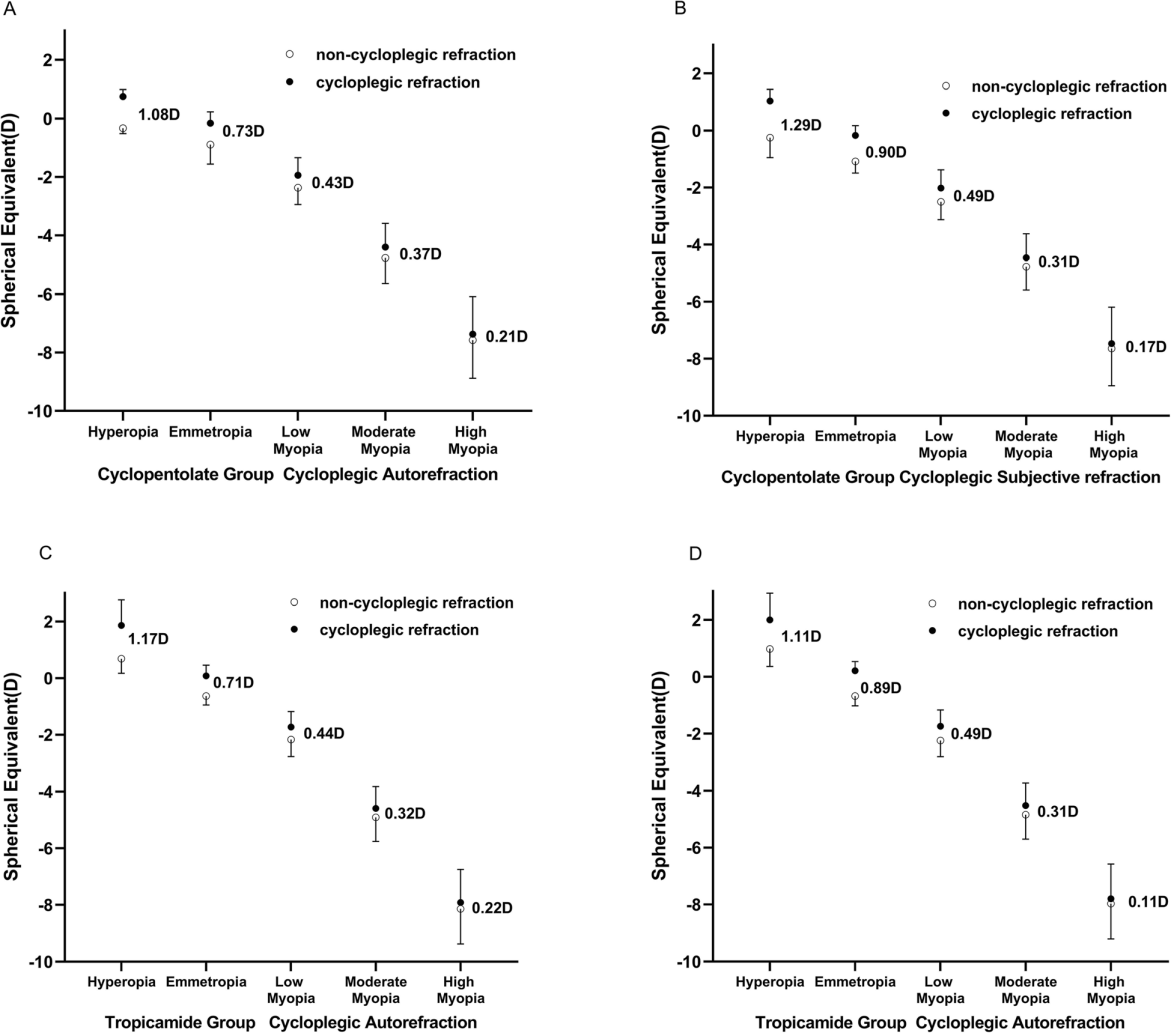
Distribution of non-cycloplegic and cycloplegic SE. The values (mean ± SD) of the non-cycloplegic and cycloplegic SE in different refractive groups for Cyclopentolate Group autorefraction (**A**), Cyclopentolate Group subjective refraction (**B**), Tropicamide Group autorefraction (**C**) and Tropicamide Group subjective refraction (**D**). Hyperopia: SE > + 0.50 D; Emmetropia: − 0.5D < SE ≤ + 0.5D; Low Myopia: − 3.0D < SE ≤ − 0.5D; Moderate Myopia: − 6.0D < SE ≤ − 3.0D; High Myopia: SE ≤ − 6.0 D; D: Diopter

7Figure 1 shows the distributions of non-cycloplegic and cycloplegic SE. All these differences were statistically significant (all *p* < 0.05). As shown in Fig. 1, young adults were divided into different categories by cycloplegic SE and difference in SE before and after cycloplegia were compared among the five categories in both groups. In the cyclopentolate group, the difference in autorefraction SE after cycloplegia decreased from 1.08 ± 0.70D in hyperopia to 0.21 ± 0.28D in high myopia. In the tropicamide group, the difference in autorefraction SE after cycloplegia decreased from 1.17 ± 0.73D in hyperopia to 0.22 ± 0.19D in high myopia. Changes in autorefraction SE after cycloplegia were similar between the cyclopentolate group and the tropicamide group (0.39 ± 0.36D vs 0.39 ± 0.40D, *p* > 0.05). Spearman correlation showed a significant positive correlation between difference in SE before and after cycloplegia and cycloplegic refraction in both the cyclopentolate group (*r* = 0.40, *p* < 0.01) and the tropicamide group (*r* = 0.54, *p* < 0.01). The slope of the regression line was 0.059 in the cyclopentolate group and 0.063 in the tropicamide group, which indicated the more hyperopia or less myopia the larger difference in SE.

**Fig. 2:**
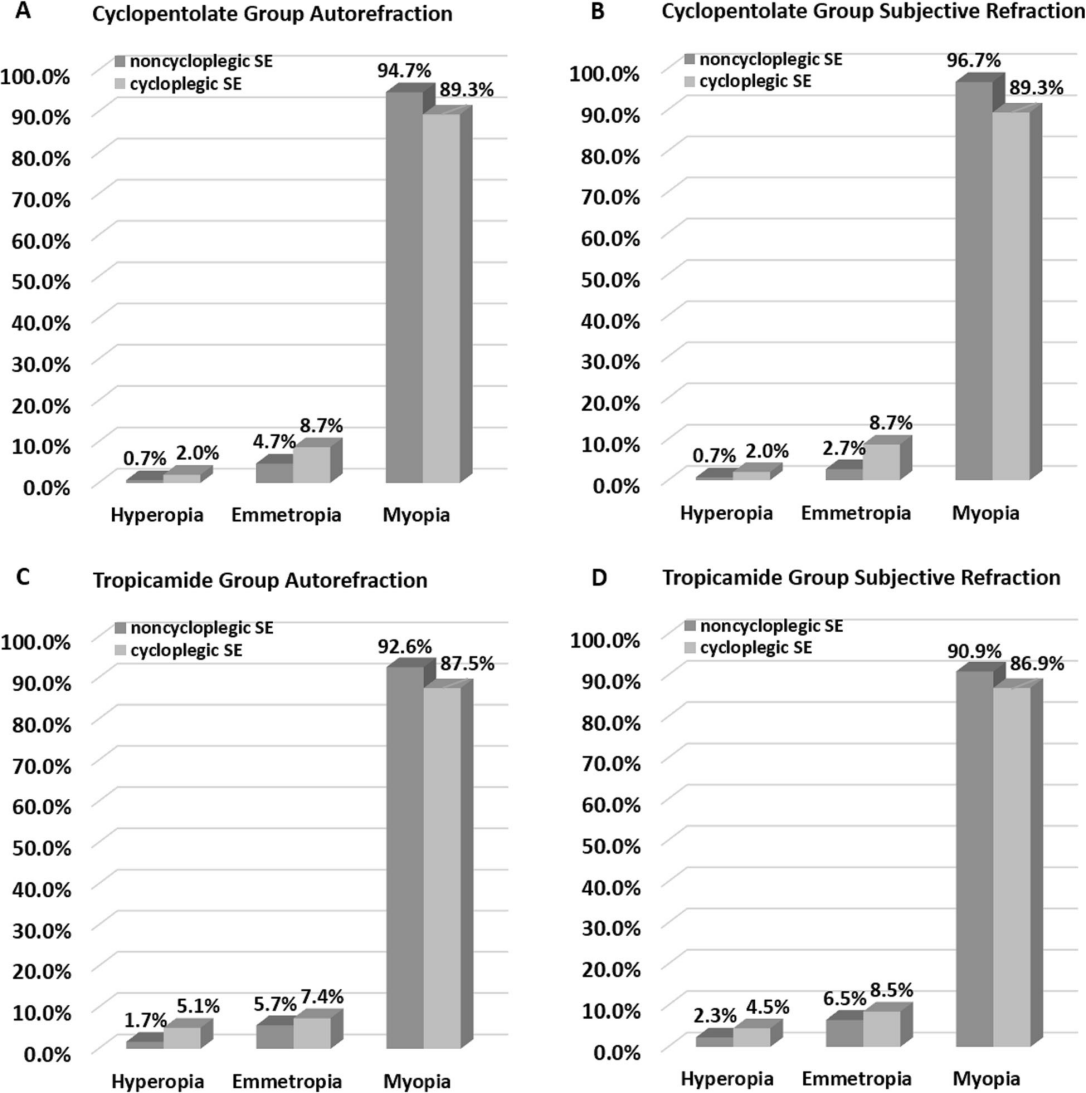
Prevalence of refractive errors based on non-cycloplegic (NC) and cycloplegic (C) data for Cyclopentolate Group autorefraction (**A**), Cyclopentolate Group subjective refraction (**B**), Tropicamide Group autorefraction (**C**) and Tropicamide Group subjective refraction (**D**). Hyperopia: SE > + 0.50 D; Emmetropia: − 0.5D < SE ≤ + 0.5D; Myopia: SE ≤ -0.50D

Figure 2 illustrates the prevalence of refractive errors based on non-cycloplegic (NC) and cycloplegic (C) SE in both groups. Before cycloplegia, the prevalence of myopia was 0.7% in the cyclopentolate group and 1.7% in the tropicamide group. After cycloplegia, the prevalence of myopia was 2.0% in the cyclopentolate group and 5.1% in the tropicamide group.

## Discussion

Our results indicated that the values of SE and sphere component were significantly different before and after cycloplegia in both the tropicamide group and the cyclopentolate group. This suggests that it is necessary to perform cycloplegia refraction for Chinese young adults to obtain accurate refractive errors. No significant difference was found between autorefraction and subjective refraction with cycloplegia (*p* > 0.05). The cycloplegic effects of the two cycloplegic agents is comparable to each other. The value of cylinder component before and after cycloplegia was not significantly different in our study.

Our results indicated that cycloplegic refractions were generally more positive or less negative than non-cycloplegic refractions in the cyclopentolate group and the tropicamide group. Our results were consistent with the previous studies [10–12, 26]. Mimouni et al [10] reported 700 soldiers aged 18 to 21 years using 1% cyclopentolate and found the difference in SE was 0.46 D in myopes and 1.30 D in hyperopes. They concluded that it was necessary to perform cycloplegia in this age group (18–21 years). The Tehran Eye study [12] analyzed participants with a wide age range from 5 to 95 years and showed that the mean difference in SE after using 1% cyclopentolate was around 0.4D in the 16–20 age group. In the study of Krantz, they used 1% tropicamide as cycloplegic agent and showed the difference in SE for participants (aged 22–39 years) was 0.44D [11]. Another study of 7793 healthy young adults (mean aged 20.2 ± 1.5 years) was conducted to compare autorefractions before and after cycloplegia [9]. The difference in SE with a mean of 0.83 ± 0.81D (median 0.63D) is larger than our findings [9]. First, the distribution of cycloplegic refractions in population was different between their study and ours. Second, their study was based on a cycloplegic regimen of two drops of 1% cyclopentolate followed by one drop of 0.5% tropicamide. Only cyclopentolate or tropicamide was used in our study. Those two factors may explain the different findings.

We also found that more positive or less negative cycloplegic refraction was associated with the higher difference in SE, which was consistent with previous studies in children or young adults [8, 10, 12, 13]. Hyperopes showed a larger difference in refraction before and after cycloplegia than myopes (1.08 ± 0.70D vs 0.35 ± 0.31 D in cyclopentolate group and 1.17 ± 0.73D vs 0.32 ± 0.26D in tropicamide group, *p* < 0.05 for all). It was speculated that accommodation capacity was stronger in hyperopes than in myopes. In the study of Sanfilippo, they reported that hyperopes aged 13 to 26 years tend to exhibit greater differences in refraction after cycloplegia than myopes [13]. The Tehran Eye Study [12] reported that in the < 25 years age group, the difference in SE between cycloplegic and noncycloplegic refractive errors was higher for cycloplegic hyperopes (0.65D), than for cycloplegic emmetropes (0.30D), and cycloplegic myopes (0.17D) (*p* < 0.001). The smallest difference in SE was for eyes with high myopia. The difference of SE value before and after cycloplegia was statistically significant in high myopes, with a mean value of 0.21D in cyclopentolate group and 0.22D in tropicamide group, which was of no clinical significance.

The findings of our study showed that there was no significant difference between autorefraction and subjective refraction with cycloplegia. In the cyclopentolate group, the mean difference in SE between autorefraction and subjective refraction after cycloplegia was 0.03D (*p* > 0.05). In the tropicamide group, the mean difference in SE between autorefraction and subjective refraction after cycloplegia was 0.06D (*p* > 0.05). Choong et al [27] found that there was a tendency of over minus correction when the autorefractors were used under noncycloplegic conditions. No significant difference was found in mean SE between autorefraction and subjective refraction after cycloplegia. The Tehran Eye Study [28] reported that mean difference between cycloplegic autorefraction and subjective refraction was 0.62 ± 0.54 D (*p* < 0.001) for participants with a mean age of 31.7 years (range 5–95 years) and inter-method differences significantly decreased with age (*p* < 0.001). There are two factors that could explain the differences in results with our study. First, their study was conducted on participants with age range of 5–95 years, which has a wider age range than ours. Second, subjective refraction was measured under noncycloplegic condition which contributes to more negative or less positive than cycloplegic refraction. We suggested that autorefraction provides an alternative method used in place of subjective refraction in Chinese young adults under cycloplegic conditions in epidemiological studies of refractive errors.

Our study confirmed that cyclopentolate had no statistically significant superiority in cycloplegia efficacy compared with tropicamide. This result is consistent with several studies that compare cyclopentolate to tropicamide on the basis of refraction results [20–22]. In the study of 28 myopic adult refractive surgery patients (mean aged 35.4 years) in California [21], they reported that there is no statistically significant difference between tropicamide and cyclopentolate cycloplegic refractions. The study published in 1961 [22], demonstrated that cyclopentolate and tropicamide reduced accommodation to a similar level, but accommodation recovered much more quickly with tropicamide. Ihekaire et al [20] found that the cycloplegic effect of cyclopentolate was stronger than tropicamide in 25 Black young adults aged 17 to 29 years with dark irises. The epidemiological refraction examination of young adults requires a rapid, safe, effective method of obtaining accurate refractive errors. The cycloplegic effects of two cycloplegic agents were similar for Chinese young adults with dark irises. Because of rapid onset cycloplegic effect and shorter duration of peak effect, we suggested that tropicamide can be considered as a viable substitute for cyclopentolate in refraction study of Chinese young adults.

Our research had several limitations. We performed cycloplegic refraction until the pupils are fully dilated in our study. However, there are some studies that found that the time of maximum cycloplegia was earlier than that of maximum mydriasis [18, 29]. Thus, the time of the cycloplegic refraction performed may also vary from study to study.

## Conclusions

In conclusion, cycloplegic measurements were generally more positive or less negative than non-cycloplegic refractions. It is necessary to perform cycloplegia refraction for Chinese young adults with dark irises to obtain accurate refractive errors. We suggest that cycloplegic autorefraction using tropicamide may be considered as a reliable method for epidemiological studies of refraction in Chinese young adults with dark irises.

## Data Availability

Data and materials are available upon request from the corresponding author at rwei@tmu.edu.cn.
